# Broomrape Species Parasitizing *Odontarrhena lesbiaca* (Brassicaceae) Individuals Act as Nickel Hyperaccumulators

**DOI:** 10.3390/plants10040816

**Published:** 2021-04-20

**Authors:** Panayiotis G. Dimitrakopoulos, Maria Aloupi, Georgios Tetradis, George C. Adamidis

**Affiliations:** 1 Biodiversity Conservation Laboratory, Department of Environment, University of the Aegean, 811 00 Mytilene, Greece; gtetradis@outlook.com; 2 Water and Air Quality Laboratory, Department of Environment, University of the Aegean, 811 00 Mytilene, Greece; malou@aegean.gr; 3 Community Ecology Division, Institute of Ecology and Evolution, University of Bern, 3012 Bern, Switzerland; georgios.adamidis@iee.unibe.ch

**Keywords:** *Alyssum lesbiacum*, biotic interaction, metallophytes, *Phelipanche nana*, *Phelipanche nowackiana*, plant parasites, serpentine soils, ultramafic

## Abstract

The elemental defense hypothesis supports that metal hyperaccumulation in plant tissues serves as a mechanism underpinning plant resistance to herbivores and pathogens. In this study, we investigate the interaction between *Odontarrhena lesbiaca* and broomrape parasitic species, in the light of the defense hypothesis of metal hyperaccumulation. Plant and soil samples collected from three serpentine sites in Lesbos, Greece were analyzed for Ni concentrations. *Phelipanche nowackiana* and *Phelipanche nana* were found to infect *O. lesbiaca*. In both species, Ni concentration decreased gradually from tubercles to shoots and flowers. Specimens of both species with shoot nickel concentrations above 1000 mg.kg^−1^ were found, showing that they act as nickel hyperaccumulators. Low values of parasite to *O. lesbiaca* leaf or soil nickel quotients were observed. *Orobanche pubescens* growing on a serpentine habitat but not in association with *O. lesbiaca* had very low Ni concentrations in its tissues analogous to excluder plants growing on serpentine soils. Infected *O. lesbiaca* individuals showed lower leaf nickel concentrations relative to the non-infected ones. Elevated leaf nickel concentration of *O. lesbiaca* individuals did not prevent parasitic plants to attack them and to hyperaccumulate metals to their tissues, contrary to predictions of the elemental defense hypothesis.

## 1. Introduction

Parasitic symbiosis describes a type of interspecific interaction in which the host organism is harmed while the parasite receives a benefit [1]. Parasitic plants attack either roots or shoots (and rarely leaves; [2]) of their hosts using specialized structures named haustoria [3], which enable them to obtain nutrients, water, and carbohydrates through a connection with the vascular system of their host [4,5,6]. The role of parasitic plants in ecosystems seems to be crucial, as they are considered to affect ecosystem structure and functioning [7,8]. Parasitic plants present high diversity levels as about 4500 species (belonging to 20 families) have been identified, which represent about 1% of all known angiosperms worldwide [9,10].

Broomrapes are obligate non-photosynthetic holoparasitic root plants belonging to the genera *Orobanche* and *Phelipanche* (Orobanchaceae) [11,12,13], whose native distribution range mostly extends to the temperate zone of the northern hemisphere [14]. Broomrapes are estimated at around 150–200 species [15], with no direct economic importance, but with devastating consequences to infected crops (e.g., legumes, vegetables, Brassica crops) mainly through yield reductions [9].

Serpentine habitats are characterized by adverse environmental conditions for plant growth [16,17] due to their physical and chemical features. They are often rocky, vulnerable to erosion, with small amounts of organic matter, and reduced moisture retention capacity [18,19]. However, plant survival in these habitats is mostly influenced by soil chemistry. There are three main reasons jointly referred to as “serpentine syndrome”: (a) low calcium to magnesium ratio, (b) high levels of heavy metals, and (c) a lack of essential macronutrients for plants [18,19]. Plant survival, growth, and reproduction are possible in these environments through the development of adaptation strategies, such as metal hyperaccumulation [20,21].

A total of 721 plant species have been reported in the literature to hyperaccumulate at least one metal or metalloid, such as As, Cd, Co, Cu, Cr, Mn, Ni, Pb, Se, Tl, and Zn, while about 532 of them have been identified as Ni hyperaccumulators [22], i.e., plants in which Ni concentration higher than 1000 mg.kg^−1^ has been recorded in above-ground tissues of at least one specimen in their natural environment [23,24,25]. In addition, 85–90% of Ni-hyperaccumulators are endemic to serpentine (ultramafic) soils [26]. The genus *Odontarrhena* (syn. *Alyssum*, Brassicaceae) includes 62 hyperaccumulator taxa of which 48 are Ni hyperaccumulators [22,27]. *Odontarrhena lesbiaca* P. Candargy (≡ *Alyssum lesbiacum* (P. Candargy) Rech. f.) is a micro-edaphic endemic species of serpentine soils of the island of Lesbos, Greece [28,29] and highly capable of hyperaccumulating Ni in its aboveground parts [30,31,32,33,34].

Several hypotheses have been formulated to provide possible explanations for the plant hyperaccumulation trait evolution associated with the ability to survive, grow, and reproduce in serpentine environments [19]. The elemental defense hypothesis [35] is the most established one, supporting that toxic metal hyperaccumulation in plant organs and tissues serves as a mechanism underpinning plant resistance to herbivorous insects [36,37] or slugs [38], and pathogens (fungi or bacteria; [39,40]). Comprehensive reviews of experiments or studies testing elemental defense hypothesis indicate that elevated Ni levels in plant tissues act defensively against herbivores -depending on their feeding mode- or pathogens [41,42], though the opposite results have also been found [41]. In accordance with this hypothesis, Ni hyperaccumulation could defend plants against attacks by plant parasites [43].

The present study aims to investigate the interaction between *O. lesbiaca* and broomrape species in the island of Lesbos, Greece. More specifically, the objectives of the present study are: (a) to record broomrape species parasitizing *O. lesbiaca*; (b) to detect whether parasitic plants receive nickel from their host, and to identify possible differences in Ni content between different plant parts and populations of broomrape species; (c) to examine potential relationships between nickel concentration in broomrapes, *O. lesbiaca* and soil; and (d) to identify differences in Ni levels between infected and non-infected individuals of *O. lesbiaca*.

## 2. Results

### 2.1. Broomrape Species Recorded

Three broomrape species, *Phelipanche nana* (Reut.) Soják, *Phelipanche nowackiana* (Markgr.) Soják and *Orobanche pubescens* d’Urv., were identified in serpentine soils sampled in Lesbos, but only two of them (*P. nana* and *P. nowackiana*) were found to infect *O. lesbiaca*. *P. nana* was recorded in all three study sites (Loutra, Ampeliko, Olympos), *P. nowackiana* was present only in Olympos, while *O. pubescens* was found only in Loutra, but not in association with *O. lesbiaca*. However, *O. pubescens* was also found in a non-serpentine soil in Loutra.

### 2.2. Ni Concentration in Broomrape Species

**Figure 1:**
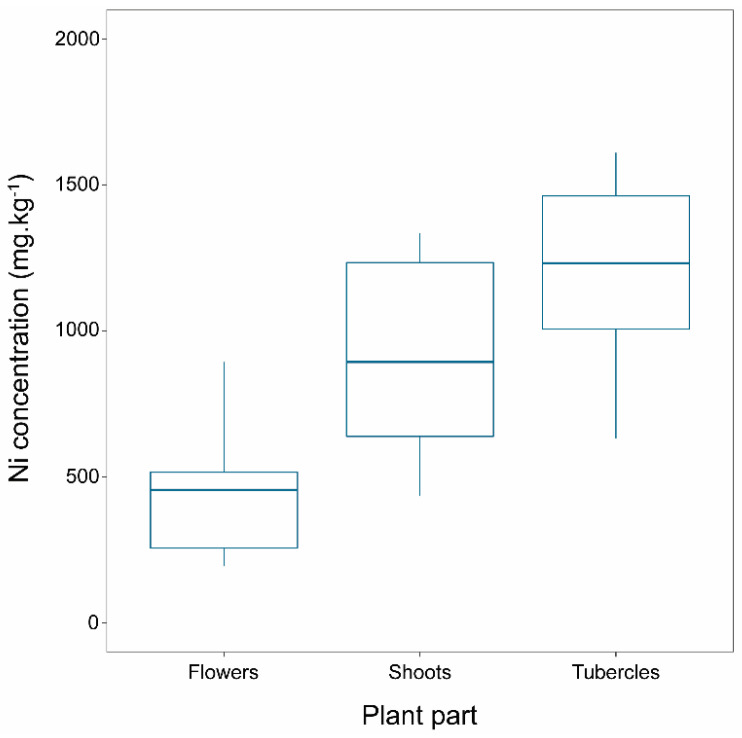
Ni concentration (mg.kg^−1^) in different parts (flower, shoot, and tubercle) of *Phelipanche nowackiana* individuals collected from Olympos, Lesbos, Greece. The central horizontal line in the box plots represents the median of the samples; the box plot edges represent the first and third quartile. The interquartile range (IQR) within the boxes present the central 50% of the values. The whiskers show the range of observed values and the locations of the minimum and the maximum values.

Nickel concentration in *P. nowackiana* individuals differed significantly among different plant parts (F_2,21_ = 9.165, *p* = 0.001). Ni concentration in flowers was statistically lower than that of the tubercles (*p* = 0.001). In contrast, shoot concentrations were intermediate and did not differ statistically neither from those of flowers (*p* = 0.092) nor of tubercles (*p* = 0.121). Mean Ni concentration in tubercles was far above 1000 mg.kg^−1^, while in shoots it was very close to that threshold, with some specimens exceeding it (Table 1; Figure 1).

**Figure 2:**
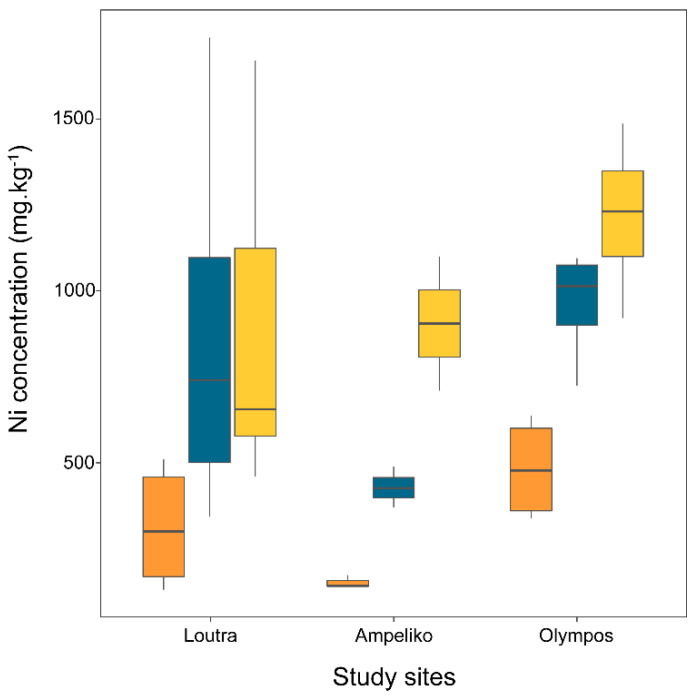
Ni concentration (mg.kg^−1^) in different parts (flowers: orange boxplots; shoots: blue boxplots; tubercles: yellow boxplots) of *Phelipanche nana* individuals collected from three serpentine sites (Loutra, Ampeliko, Olympos) in the island of Lesbos, Greece. The central horizontal line in the box plots represents the median of the samples; the box plot edges represent the first and third quartile. The interquartile range (IQR) within the boxes present the central 50% of the values. The whiskers show the range of observed values and the locations of the minimum and the maximum values.

No significant interaction between site and plant part on Ni concentration was observed for *P. nana* individuals (Figure 2). The main effect of site on Ni concentration was not significant at a = 0.05 (*p* = 0.064), but a statistically significant variation was observed in the Ni concentration of the different parts (F_2,32_ = 10,537, *p* < 0.001). The average Ni concentration of the flowers differed significantly from those of the shoots (*p* = 0.007) and tubercles (*p* < 0.001), while the mean concentration values of shoots and tubercles did not differ significantly (*p* = 0.477). Mean values of Ni concentration in tubercles were close to or above 1000 mg.kg^−1^ at all study sites as well as in shoots at two of the sites (Loutra, Olympos) (Table 1, Figure 2).

Ni concentration in different parts of *O. pubescens* across different soil types was very low (all values less than 30 mg.kg^−1^; see Table 1). No significant interaction between soil type and plant part on Ni concentration was observed for *O. pubescens* individuals at a = 0.05 (F_2,16_ = 3.221, *p* = 0.067). Plants growing on the serpentine soil type had higher Ni concentrations in their tissues relative to their conspecifics on the non-serpentine one (F_1,16_ = 25.867, *p* < 0.001), while plant parts differed in their Ni concentration (F_2,16_ = 3.795, *p* = 0.045; post hoc test did not reveal a clear pattern in Ni distribution among the plant parts).

### 2.3. O. lesbiaca—Broomrape Species Complex

Parasite/*O. lesbiaca* leaf Ni quotients were low (Table 2). No significant interactive effects of site and plant part nor of plant part and species emerged (*p* > 0.05). The quotient of broomrape plant part Ni concentrations to these of *O. lesbiaca* was higher in tubercles compared to flowers (F_2,19_ = 7.956, *p* = 0.003). Focusing on *P. nana*, the species present at all three sites, the quotient was significantly higher in tubercles followed by shoots and flowers (F_2,14_ = 16.238, *p* < 0.001). Soil total nickel concentration did not have a significant effect (*p* > 0.05 in all cases) when added as a covariate in the initial ANOVA models.

Nickel parasite/soil quotients were also low (Table 2), with tubercles presenting significantly higher quotients relative to the other two plant parts (F_2,19_ = 10.902, *p* = 0.001). Broomrape-to-total soil nickel concentration quotients appear to be significantly higher in Olympos relative to those from Ampeliko (F_2,19_ = 10.902, *p* = 0.001). No significant interactive effects between site and plant part and plant part and species emerged (*p* > 0.05).

### 2.4. Effects of Broomrapes on Leaf Nickel Concentration of O. lesbiaca

**Figure 3:**
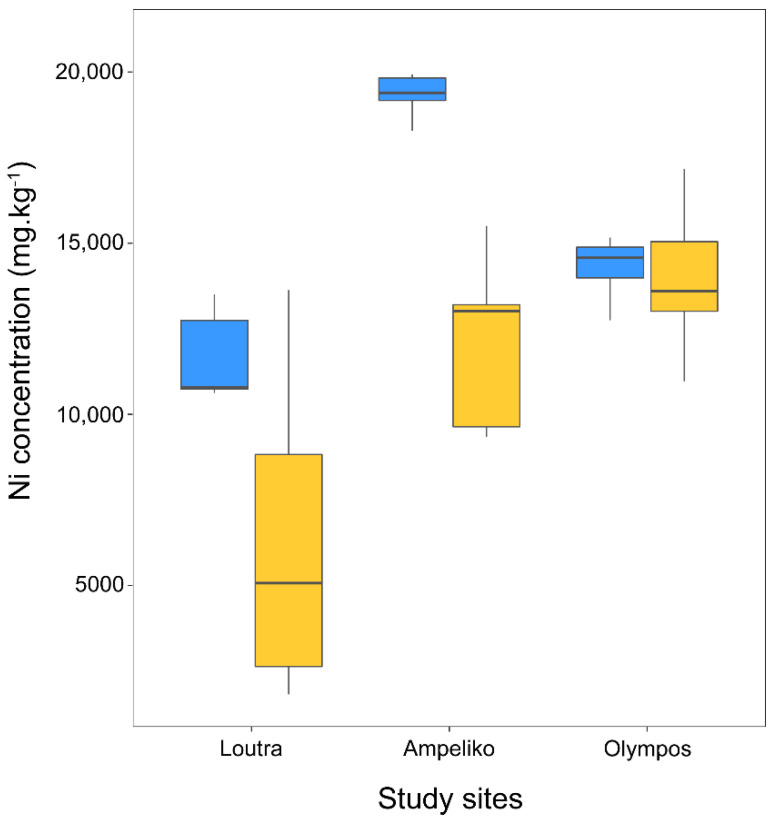
Leaf Ni concentration (mg.kg^−1^) of healthy (blue boxplots) and infected (yellow boxplots) *Odontarrhena lesbiaca* individuals from three serpentine sites (Loutra, Ampeliko, Olympos) in the island of Lesbos, Greece. The central horizontal line in the box plots represents the median of the samples; the box plot edges represent the first and third quartile. The interquartile range (IQR) within the boxes present the central 50% of the values. The whiskers show the range of observed values and the locations of the minimum and the maximum values.

Leaf Ni concentration of infected *O. lesbiaca* was significantly lower compared to non-infected individuals (F_1,38_ = 25.810, *p* < 0.001). Furthermore, a significant ‘site x infection’ interaction on leaf nickel concentration of *O. lesbiaca* was detected (F_2,38_ = 5.373, *p* = 0.009), meaning that differences between sites affected the differentiation in leaf nickel concentration across infected vs. non-infected individuals (Figure 3). More specifically, leaf Ni concentration of infected *O. lesbiaca* individuals was lower in Loutra and Ampeliko compared to their non-infected conspecifics (*p* < 0.05 in all cases), while a similar but non-significant trend was found in Olympos (*p* > 0.05). On average, leaf nickel concentrations in both infected and non-infected individuals of *O. lesbiaca* were significantly lower in Loutra relative to Ampeliko and Olympos (i.e., L < A, O based on pairwise comparisons among the estimated marginal means; *p* < 0.001 in all cases).

## 3. Discussion

The elemental defense hypothesis has not been confirmed in the few host–parasite interaction studies where a hyperaccumulating species served as the host. The ability of *Streptanthus polygaloides* to hyperaccumulate nickel did not prevent *Cuscuta californica* attack [43], and the same is true for the infection of *Alyssum murale* (syn *Odontarrhena chalcidica*) by *Orobanche nowackiana* (syn. *Phelipanche nowackiana*) [44]. In accordance with these studies, broomrape species infecting individuals of *O. lesbiaca* appear to be resistant to increased nickel concentrations.

*Phelipanche nowackiana* is a rare parasitic flowering plant restricted to European serpentine soils [45] which infects species belonging to genera *Odontarrhena* and *Bornmuellera* in Albania, Greece, and Turkey [46]. The first record of *P. nowackiana* in Lesbos comes from 1988, when during an expedition, it was reported that it probably infects *O. lesbiaca* individuals, on the basis of the two species growing in its proximity [46]. The occurrence of this species in Lesbos was recently confirmed in Olympos, in a different locality at a higher elevation than the first record [47]. In our study, we found specimens of the species having nickel concentrations above 1000 mg.kg^−1^ in their aboveground tissues (shoots) (Table 1), thus meeting the definition of nickel hyperaccumulation [23,25]. Shoot nickel concentration was higher than that of the aboveground part of the only specimen of the species previously collected by Reeves [23] (616 mg.kg^−1^) in Lesbos. In our study, *P. nowackiana* shoot concentrations were also much higher than the ones measured in the same species parasitizing *Alyssum murale* individuals in Albania (299 mg.kg^−1^ in leaves and 184 mg.kg^−1^ in shoots on average; [44]), as well as in non-hyperaccumulating species (containing less than 100 mg.kg^−1^ in their tissues; [31]) growing in the same habitat. Our results confirm the close association between *P. nowackiana* and *O.lesbiaca*, and demonstrate the ability of *P. nowackiana* to hyperaccumulate metals in its above- and below-ground tissues, contrary to the predictions of the elemental defense hypothesis [41].

*Phelipanche nana* is parasitic to a variety of host plant species [48]. Our results showed that it contains markedly higher nickel in aboveground parts relative to other plant species thriving in the study sites and considered as excluders [31]; most importantly, it was found able to hyperaccumulate nickel in its shoots in two out of three of its distribution sites (Table 1). Its tissue nickel concentrations were comparable with those of *P. nowackiana* in Olympos where the two species coexisted, and higher than those demonstrated by other parasites of nickel hyperaccumulating species [43,44]. To the best of our knowledge, this is the first study to show that this species acts as a nickel hyperaccumulator.

Our results indicate that in both *P. nowackiana* and *P. nana*, Ni concentration decreased gradually from tubercles to shoots and flowers, with the latter demonstrating the lowest Ni concentration values. This finding is in accordance with the hypothesis that within Ni-hyperaccumulating plants there is a gradation of filtering out Ni, from leaves to tissues related with the flower-pollinator interface, which was interpreted as a “corrective” adaptation mechanism related to the hyperaccumulator-pollinator interaction [33]. This filtering is also supported by the low Ni-concentrations recorded on the anthers and pollen of *O. nowackiana* in Albania [49].

*Orobanche pubescens* grew on a serpentine habitat but not in association with *O. lesbiaca*. This species had very low concentrations in both above- and below-ground tissues (<30 mg.kg^−1^), demonstrating its ability to tolerate serpentine habitat conditions [19], and especially high soil nickel concentrations. Even so, individuals growing on serpentine soil had higher Ni in all their parts compared to those collected in a non-serpentine soil type, similarly to the trend recorded in other non-hyperaccumulating plants growing on both serpentine and non-serpentine habitats in Lesbos [31]. Ni uptake by non-hyperaccumulators was shown to mainly occur via the low-affinity transport systems of other bivalent micronutrient elements, e.g., Zn, Fe, and Cu [50]. Thus, higher Ni in tissues of these plants when occurring in serpentine soils could be well justified. On the other hand, the low Ni concentration in *O. pubescens* suggests that nickel is absorbed through its hosts and not directly from the serpentine soil [43].

In general, *Orobanche* and *Phelipanche* spp. obtain all necessary minerals from their hosts, through connections with both xylem and phloem [51], and not by taking them up from the soil through their root system [2]. The lack of a relationship between soil Ni and parasitic plant Ni levels assessed in this study and elsewhere [43] favors this conclusion. Hyperaccumulating plants have evolved highly efficient physiological mechanisms for taking up Ni in their roots and for subsequently translocating and sequestrating it into their aerial parts. Ni upwards flow through xylem has been well documented in a number of hyperaccumulators and in many cases Ni speciation in the xylem sap has been estimated as well [50]. In *O. lesbiaca* as well as in other hyperaccumulator species of the genus *Odontarrhena* selective chelation mainly with histidine was revealed to be involved in Ni translocation from root to shoot and finally to leaves via xylem although ca. 50% of the metal was estimated to be in the free hydrated ionic form (Ni^2+^) [52]. Nonetheless, although long overlooked, phloem translocation has more recently been demonstrated in both woody tropical and in herbaceous hyperaccumulating plant species [50]. Although phloem transport has not been proven in *O. lesbiaca*, the high Ni levels recorded in the flowers of this species (way above 1000 mg.kg^−1^; [32,33]) provide supportive evidence to this process since flowers are the main sink of phloem translocation [50]. In line with this hypothesis, Bani et al. [44] concluded that *P. nowackiana* should obtain nutrients exclusively from the phloem of *A. murale* given that its low transpiration rate could not account for nutrient uptake via diverting the flow of xylem from the host [53].

Despite the high Ni concentrations in tubercles and aboveground parts of both *P. nowackiana* and *P. nana*, only a small amount of Ni accumulated in the host plant finds its way into the parasite, as shown by the low values of the quotient of Ni concentration in broomrape species’ parts versus Ni concentration in *O. lesbiaca* leaves (Table 2). A similar finding in *C. californica* parasitizing *S. polygaloides*, the quotient being higher in that case (0.25 on average) though, was attributed to the inability of the parasite to more effectively exclude metals from its tissues [43].

A higher nickel concentration in tissues of parasites attacking *O. lesbiaca* individuals relative to those infecting *A. murale* [44] may express the higher nickel hyperaccumulation capacity recorded in *O. lesbiaca* [31,32]. However, the relationship between *O. lesbiaca* and parasite Ni levels as expressed through parasite-to-*O. lesbiaca* nickel concentration quotients could not be attributed to soil nickel concentrations.

Infected *O. lesbiaca* individuals showed lower leaf nickel concentrations relative to non-infected ones, as in the case of *A. murale* [44], but this effect was mediated through the site effect. This probably indicates that other factors related to site characteristics such as important soil elemental concentrations, soil porosity and pH, or rainfall levels [24] could contribute to explaining this pattern. Infection by parasites also altered the gradient in leaf nickel concentrations we have observed in previous studies in *O. lesbiaca* individuals across study sites [31,32]. While healthy plants growing in Ampeliko always showed higher leaf nickel concentration relative to those from Olympos and Loutra (L < O < A) following the soil Ni concentrations, the Ni concentrations of infected plants in Loutra were the only ones differing from the other two sites (L < O, A).

The negative effect of parasites on leaf nickel concentration of *O. lesbiaca* demonstrates that this could potentially decrease nickel phytoextraction yield, as in the case of *A. mulare* [44]. However, a limitation of our study is that we did not measure shoot nickel concentration, nor did we calculate differences in biomass production between healthy and infected individuals of different phenological stages.

In conclusion, elevated leaf nickel concentration of *O. lesbiaca* individuals did not prevent *Phelipanche* parasitic plants to attack them and to hyperaccumulate metals in their tissues, contrary to the predictions of the elemental defense hypothesis. The parasitic attack significantly lowers the hyperaccumulated leaf Ni concentration in two out of three studied populations of *O. lesbiaca*, potentially affecting its Ni phytoextraction capacity.

Future studies investigating the effect of parasites on nickel phytoextraction yield of *O. lesbiaca* will require the establishment of plots with both healthy and infected individuals in each site, while at the same time standardizing the age and density of *O. lesbiaca* individuals as well as the density and/or the richness of parasites on the infected plots. Although our results indicate that parasite-free environments would probably be more efficient for agromining, a better understanding of the mechanisms underlying interactions between metal hyperaccumulating and parasitic plants would provide great insights for the success of these technologies.

## 4. Materials and Methods

### 4.1. Study Area and Species

Three serpentine sites (Loutra, Olympos, Ampeliko) were selected in the eastern Mediterranean island of Lesbos, Greece [31,32,54]. Loutra (N 39°02′36.8″, E 026°32′55.0″; 94 m a.s.l.) is a lower elevation site in the eastern part of Lesbos while Ampeliko (N 39°05′46.4″, E 026°19′59.9″; 361 m a.s.l.) and Olympos (N 39°04′33.3″, E 026°20′16.3″; 759 m a.s.l.) are in the central part of the island and at higher elevations (see [31] for a detailed description of the sites). All sites are openings in pine forest (*Pinus brutia* Ten.). Soil nickel concentration is significantly higher at Ampeliko site (3326 ± 202 mg.kg^−1^) relative to Olympos (1948 ± 113 mg.kg^−1^) and Loutra (1197 ± 16.8 mg.kg^−1^) [31]. These serpentine sites were selected based on the presence of extensive populations of *O. lesbiaca*. *O. lesbiaca* was highly dominant in all sites, contributing a large part of the aboveground biomass: up to ca. 50% at Loutra, more than 85% at Ampeliko and more than 95% at Olympos [54,55].

Our sampling aimed at locating *O. lesbiaca* individuals infected by broomrapes. However, at one site, Loutra, we found another species of broomrape on serpentine soil but not in association with *O. lesbiaca*. To test for differences in nickel concentration between broomrape individuals of this species on serpentine and on non-serpentine soil, we collected individuals of the same species in an adjacent non-serpentine habitat (soil Ni concentration: 45.3 ± 2.5 mg.kg^−1^). To establish an unbiased comparison, the sampled broomrape individuals parasitised the same host species in both soil types.

### 4.2. Plant Sampling

Plant sample collection took place in late April–early May of 2018. Broomrape species were recorded and identified after two sampling cycles at each site [47,56,57]. Sampling for the host and parasites took place concurrently at each site. Flowering individuals of broomrape species were collected. *O. lesbiaca* has a four-year life cycle [32], and plants of all phenological stages were present at each site. During our host sampling, we focused on three-year-old individuals both because they were the most infected by parasites, and because they have been found to possess the maximum Ni phytoextraction capacity [32].

Broomrape individuals were randomly collected in each site, using a digging tool. Attention was paid to preserve their belowground part intact. During sampling, broomrape individuals were matched to their associated/nearby *O. lesbiaca* individuals in the field. To verify the process of identifying these interactions, ten additional *O. lesbiaca*-broomrape pair samples were collected in Ampeliko and Olympos sites. In these cases, whole soil blocks were sampled, soil particles were carefully removed, and the host-parasite relationship was confirmed. In each sampled pair, soil samples were also collected at a 10-cm depth inside the rhizosphere of the infected *O. lesbiaca* individuals. Leaves of healthy and infected three-year-old *O. lesbiaca* individuals were also collected at each site. All samples were stored in plastic bags for transport to the laboratory and stored at 4 °C until analysis.

### 4.3. Ni Determination in Plant and Soil Samples

In the laboratory broomrape individuals were separated into flowers, shoots including leaves, and tubercles including adventitious roots [44] to determine the distribution of Ni in the different plant parts. Small samples of individuals collected in adjacent micro-sites were pooled together to create composite samples with a mass sufficient for chemical analysis.

Before analysis, all plant parts were rinsed with ultrapure water in a filtration unit to remove any adhering soil particles. They were then pre-frozen at −25 °C and freeze-dried for 48 h in a Labconco (Kansas City, MO, USA) FreeZone 4.5 laboratory apparatus at −40 °C collector temperature under <0.1 mBar vacuum. The samples were pulverized in an agate mortar and digested with conc. HNO₃ and H₂O₂ 30% according to the USEPA’s method 3052 [58]. Soil samples were pretreated according to the NEPM Guideline on Laboratory Analysis of Potentially Contaminated Soils [59]. In brief, after extraneous components (vegetation and other non-soil material) were removed by hand, the soil samples were air-dried, sieved through a 2 mm mesh sieve, partitioned and grinded in an agate mortar. The pulverized samples were digested with conc. HNO₃, HCl, and HF followed by complexation of the residual HF with H₃BO₃ so as to assess total Ni concentrations (USEPA’s method 3052; [58]). All digestions were run in a MarsXpress system (CEM Corporation; Matthews, NC, USA). Ni determination in plant and soil samples was performed by Flame Atomic Absorption Spectrometry in a Perkin-Elmer (Waltham, MA, USA) 5100ZL spectrometer. The water used in all chemical analyses was of ultrapure grade (resistivity 18.2 MΩ·cm) provided by Merck–Millipore Milli-Q water purification system (Bedford, MA, USA) and the chemicals were of Suprapur grade supplied by Merck (Darmstadt, Germany). Sample handling in the laboratory was carried out in a Class 100 NUAIRE (NU154-524E; Plymouth, MN, USA)) laminar flow hood to avoid contamination. All Ni concentrations were expressed on a dry mass basis. To this end, residual moisture was measured after drying at 105 °C to constant mass in separate air-dried soil subsamples and digested soil sample masses were corrected as appropriate.

### 4.4. Data Analysis

For all our comparisons we used analysis of variance (ANOVA). First, we tested for differences in Ni concentration between broomrape plant parts and sites. Additionally, we examined the effect of soil type (serpentine vs. non-serpentine) on the Ni concentration of different plant parts of *O. pubescens* in those individuals that were not associated with *O. lesbiaca*. Next, we evaluated the effect of host Ni concentration to the interacting parasite, by calculating the quotient of Ni concentration in broomrape parts versus Ni concentration in *O. lesbiaca* leaves [43,60]. Broomrape parts-to-soil Ni concentration quotients were also calculated. Apart from testing for the effects of species, plant part and site, we also added soil Ni concentration as a covariate in the initial ANOVA. Finally, we investigated the effect of broomrape infection, along with the effect of site, on Ni concentration of *O. lesbiaca*. In all ANOVA designs, all two-way interaction terms were included, while post-hoc multiple comparisons were conducted using the Bonferroni correction. The ‘ggplot’ function of the ‘ggplot2’ package was used for all visualizations. All statistical analyses were conducted in R version 4.0.2 [61].

## Figures and Tables

**Table 1:** Ni concentration (mg.kg^−1^) in different plant parts (flower, shoot, and tubercle) of the three broomrape species sampled from serpentine sites (Loutra, Ampeliko, Olympos) of the island of Lesbos, Greece. Mean (±1 Standard Error, SE), minimum and maximum values are presented. Numbers in bold indicate maximum Ni concentrations exceeding the hyperaccumulation threshold (1000 mg.kg^−1^) in above-ground tissues.

		Loutra				Ampeliko				Olympos			
		Mean	SE	Min	Max	Mean	SE	Min	Max	Mean	SE	Min	Max
*P. nana*	Flower	378	110	131	956	152	11	140	174	483	76	339	638
	Shoot	859	191	343	**1737**	428	34	370	488	962	84	725	**1095**
	Tubercle	884	185	460	1670	906	196	710	1101	1218	119	921	1487
*P. nowackiana*	Flower									481	85	194	894
	Shoot									937	116	435	**1335**
	Tubercle									1417	269	631	2562
*O. pubescens* ^1^	Flower	15	5	8	25								
	Shoot	9	1	7	11								
	Tubercle	22	1	21	23								

^1^ Ni concentration (mean ± 1SE; mg.kg^−1^) in plant parts from Loutra non-serpentine habitat was: Flower: 4 ± 1; Shoot: 5 ± 1; Tubercle: 6 ± 2.

**Table 2:** Quotient of Ni concentration in broomrape species parts versus Ni concentration in *O. lesbiaca* leaves and soil sampled from two serpentine sites (Ampeliko, Olympos) of the island of Lesbos, Greece. Mean (±1 Standard Error, SE) values are presented.

		Ampeliko				Olympos			
		*Broomrape Ni/O. lesbiaca Ni*		*Broomrape Ni/Soil Ni*		*Broomrape Ni/O. lesbiaca Ni*		*Broomrape Ni/Soil Ni*	
		mean	SE	mean	SE	mean	SE	mean	SE
*P. nana*	Flower	0.014	0.002	0.040	0.001	0.034	0.012	0.176	0.047
	Stem	0.039	0.004	0.114	0.007	0.057	0.005	0.313	0.060
	Tubercle	0.075	0.014	0.194	0.026	0.081	0.013	0.430	0.025
*P. nowackiana*	Flower					0.028	0.010	0.096	0.026
	Stem					0.073	0.027	0.243	0.069
	Tubercle					0.137	0.096	0.467	0.257

## Data Availability

The data presented in this study are available on request from the corresponding author.
